# Melatonin Attenuates Pulmonary Hypertension in Chronically Hypoxic Rats

**DOI:** 10.3390/ijms18061125

**Published:** 2017-05-24

**Authors:** Ming Wai Hung, Hang Mee Yeung, Chi Fai Lau, Angela Ming See Poon, George L. Tipoe, Man Lung Fung

**Affiliations:** School of Biomedical Sciences, Li Ka Shing Faculty of Medicine, The University of Hong Kong, Pokfulam, Hong Kong SAR, China; philiphung928@hotmail.com (M.W.H.); hangmee@gmail.com (H.M.Y.); jeffery0820hk@yahoo.com.hk (C.F.L.); amspoon@hku.hk (A.M.S.P.); tgeorge@hku.hk (G.L.T.)

**Keywords:** anti-oxidant, chronic hypoxia, inflammation, lung injury, nitric oxide

## Abstract

Chronic hypoxia induces pulmonary hypertension and vascular remodeling, which are clinically relevant to patients with chronic obstructive pulmonary disease (COPD) associated with a decreased level of nitric oxide (NO). Oxidative stress and inflammation play important roles in the pathophysiological processes in COPD. We examined the hypothesis that daily administration of melatonin (10 mg/kg) mitigates the pulmonary hypertension and vascular remodeling in chronically hypoxic rats. The right ventricular systolic pressure (RVSP) and the thickness of pulmonary arteriolar wall were measured from normoxic control, vehicle- and melatonin-treated hypoxic rats exposed to 10% O_2_ for 14 days. Levels of markers for oxidative stress (malondialdhyde) and inflammation (tumor necrosis factor-α (TNFα), inducible NO synthase (iNOS) and cyclooxygenase-2 (COX-2)) and the expressions of total endothelial NO synthase (eNOS) and phosphorylated eNOS at serine1177 (ser1177) were determined in the lung tissue. We found that the RVSP and the thickness of the arteriolar wall were significantly increased in the vehicle-treated hypoxic animals with elevated levels of malondialdhyde and mRNA expressions of the inflammatory mediators, when compared with the normoxic control. In addition, the phosphorylated eNOS (ser1177) level was significantly decreased, despite an increased eNOS expression in the vehicle-treated hypoxic group. Melatonin treatment significantly attenuated the levels of RVSP, thickness of the arteriolar wall, oxidative and inflammatory markers in the hypoxic animals with a marked increase in the eNOS phosphorylation in the lung. These results suggest that melatonin attenuates pulmonary hypertension by antagonizing the oxidative injury and restoration of NO production.

## 1. Introduction

Chronic hypoxia (CH) induces pulmonary hypertension, pulmonary vascular remodeling and polycythemia in humans and experimental animals [1,2,3]. The pathophysiological processes are clinically relevant to chronic obstructive pulmonary disease (COPD) patients suffering from chronic hypoxemia [4]. Oxidative stress and inflammation are believed to be significantly involved in the pathogenic cascade of CH-induced pulmonary hypertension [5]. In fact, levels of malondialdehyde and isoprostane were reportedly elevated in COPD patients [6,7]. Antioxidant *N*-acetylcysteine ameliorated pulmonary hypertension and also vascular remodeling in experimental animals [8]. Hypoxia also induces pulmonary vascular remodeling characterized by the proliferation of smooth muscle cells, endothelial cells and fibroblasts [9]. In addition, pro-inflammatory mediators could be involved in CH-induced pulmonary hypertension because circulating tumor necrosis factor-α (TNFα) level was elevated in COPD patients [10].

Pulmonary hypertension induced by chronic hypoxia could be caused by changes in pulmonary vasoreactivity leading to an augmented vasoconstriction in responding to receptor-mediated agonists. Importantly, there are compensatory responses to chronic hypoxia, which increase the expression of endothelial nitric oxide synthase (eNOS) [11,12,13] and the endothelium-derived nitric oxide (NO)-dependent vasodilation in the pulmonary vessels [14]. It has been shown that reactive oxygen species (ROS) could increase eNOS expression transcriptionally through increasing the mRNA half-life [15] and also by Ca^2+^/CaM kinase II/janus kinase 2-dependent pathway [16]. However, the plasma NO level was reportedly lowered in COPD patients with hypoxemia, which suggests that the CH-induced eNOS upregulation could not restore the NO bioavailability [17,18].

Melatonin is a pineal hormone and also a potent free radical scavenger at physiological and pharmacological concentrations. Pharmacological doses of exogenously administration of melatonin are known to effectively antagonize oxidative injuries via its antioxidant and anti-inflammatory properties [19,20,21]. In addition, melatonin could induce endothelium-dependent vasorelaxation with an increase in the NO production mediated by elevated NOS activity [22,23]. Recent studies have shown that melatonin treatment could elevate the NO availability in the lungs of newborn sheep at high altitude [24] and also alleviate the severe pulmonary hypertension induced by monocrotaline [25] or intermittent hypoxia [26] in rats. However, it is unclear whether the melatonin treatment might ameliorate the pulmonary hypertension induced by chronic hypoxia, which is clinical relevance to the pathophysiological cascade in COPD patients. Moreover, the phosphorylation of eNOS at serine 1177 residue (ser1177) plays an important role in the activation of eNOS activities and this mechanism may be involved in the effect of melatonin on the NO availability. In this study, we examined the hypothesis that daily administration of melatonin could mitigate the pulmonary hypertension and vascular remodeling induced by chronic hypoxia via its antagonistic effects against oxidative stress and inflammation, leading to an increase in the phosphorylated eNOS (ser1177).

## 2. Results

Body weights of the hypoxic animal treated with vehicle (225.8 ± 7.3 g) or melatonin (227.1 ± 2.2 g) were lower than that of the normoxic control (259.3 ± 10.3 g). Levels of hematocrit were markedly elevated in both the vehicle- (55.9 ± 1.3%) and melatonin-treated (56.8 ± 1.6%) hypoxic groups, when compared with the normoxic control (46.4 ± 0.2%). There were no differences in the body weight or hematocrit between the vehicle- and melatonin-treated groups.

**Figure 1:**
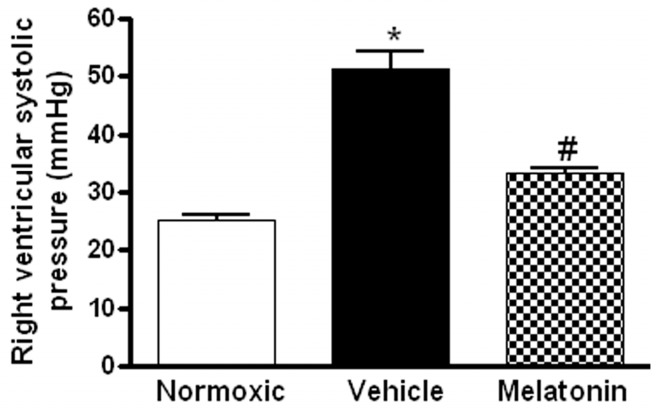
Effect of melatonin on the right ventricular systolic pressure of normoxic control and chronically hypoxic rats treated with vehicle or melatonin. *, *p* < 0.01, *n* = 6, versus normoxic group. #, *p* < 0.01, *n* = 6, versus vehicle group.

**Figure 2:**
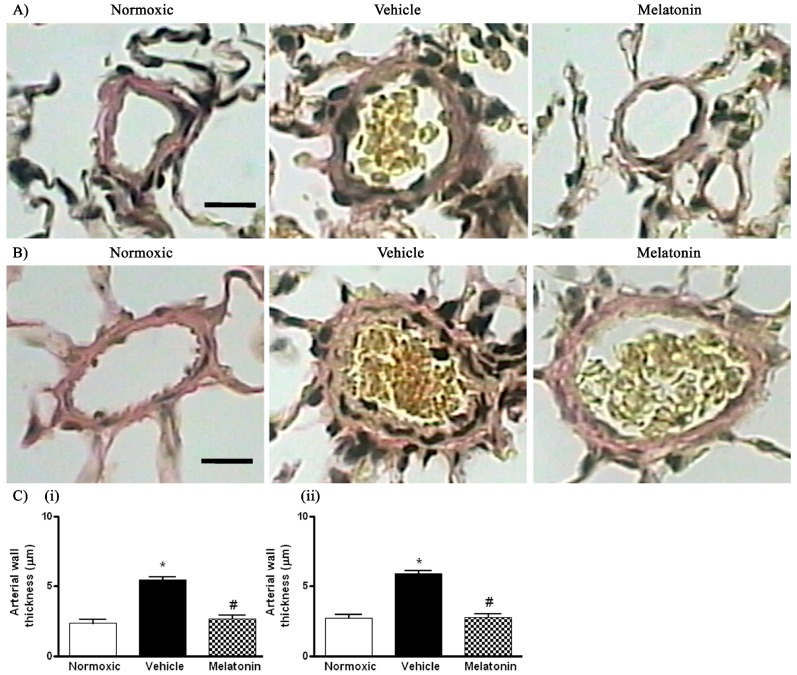
Effect of melatonin on the thickness of smooth muscle layers of pulmonary small resistance vessels in normoxic control and chronically hypoxic rats treated with vehicle or melatonin: (**A**) photomicrographs of pulmonary small resistance vessels of diameters smaller than 50 μm, scale bar = 25 μm; (**B**) photomicrographs of pulmonary small resistance vessels of diameters between 50 and 100 μm, scale bar = 25 μm and (**C**) summary of the wall thickness in vessels of diameters: (**i**) smaller than 50 μm; and (**ii**) between 50 and 100 μm. *, *p* < 0.01, *n* = 6, versus normoxic group. #, *p* < 0.01, *n* = 6, versus vehicle group.

The right ventricular systolic pressure (RVSP) was significantly elevated in the vehicle-treated hypoxic rats when compared with the normoxic control (Figure 1). The RVSP was remarkably attenuated to the normoxic level by the melatonin treatment (Figure 1). In addition, the ratio of right ventricle to left ventricle and septum weight (RV/LV+S) was significantly increased in the hypoxic rats treated with vehicle (0.48 ± 0.01) or melatonin (0.42 ± 0.02) when compared to the normoxic group (0.28 ± 0.01). Moreover, the thickness of the vascular layer of the smooth muscle cells in the pulmonary arterioles was significantly increased in the vehicle-treated hypoxic rats when compared with that of the normoxic control. The thickness of the arteriolar wall in melatonin-treated CH rats was not different from the normoxic control (Figure 2).

**Figure 3:**
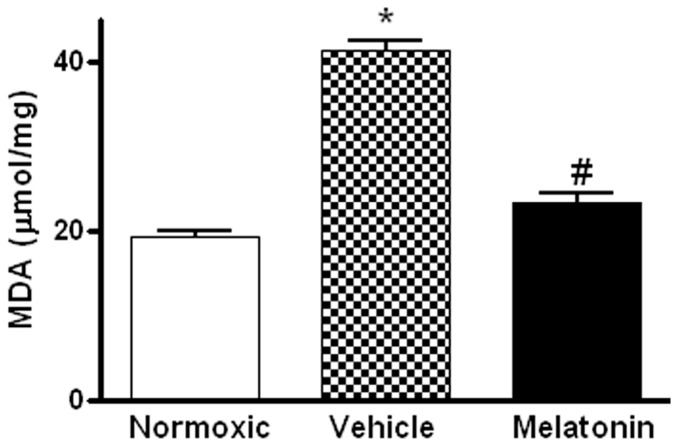
Effect of melatonin on lipid peroxidation on lung tissues of normoxic control and chronically hypoxic rats treated with vehicle or melatonin. *, *p* < 0.01, *n* = 6, versus normoxic group. #, *p* < 0.01, *n* = 6, versus vehicle group.

**Figure 4:**
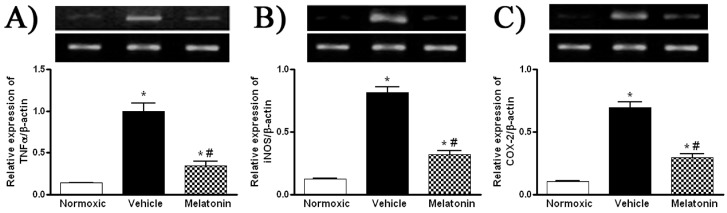
Effect of melatonin on gene expression of pro-inflammatory cytokine and inflammatory mediators: (**A**) tumor necrosis factor-α (TNFα); (**B**) inducible NO synthase (iNOS); and (**C**) cyclooxygenase-2 (COX-2), in the lung of normoxic control and chronically hypoxic rats treated with vehicle or melatonin. *, *p* < 0.01, *n* = 6, versus normoxic group. #, *p* < 0.01, *n* = 6, versus vehicle group.

Levels of MDA in the lung were significantly higher in vehicle-treated hypoxic rats than that of the normoxic control (Figure 3). The MDA level was markedly attenuated in the melatonin-treated group and was comparable to the normoxic level (Figure 3). In addition, levels of the mRNA expression of pro-inflammatory cytokine, namely tumor necrosis factor-α (TNFα), and inflammatory mediators, namely inducible NO synthase (iNOS) and cyclooxygenase-2 (COX-2), were significantly increased in the vehicle-treated hypoxic rat, whereas the expression levels were markedly reduced by the melatonin treatment (Figure 4).

**Figure 5:**
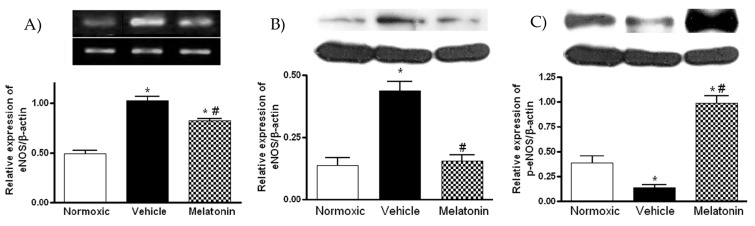
Effect of melatonin on: (**A**) gene expression of endothelial NO synthase (eNOS); (**B**) protein level of total; and (**C**) phosphorylated eNOS (ser1177) in the lung of normoxic control and chronically hypoxic rats treated with vehicle or melatonin. *, *p* < 0.01, *n* = 6, versus normoxic group. #, *p* < 0.01, *n* = 6, versus vehicle group.

The mRNA and protein levels of eNOS were significantly increased in the vehicle-treated hypoxic group (Figure 5A,B), whereas the levels were significantly lowered in the melatonin-treated group (Figure 5A,B). In contrast, the protein level of phosphorylated eNOS (ser1177) of the vehicle group was significantly lower than that of the normoxic control (Figure 5C). The level of phosphorylated eNOS was markedly elevated by the melatonin treatment (Figure 5C).

## 3. Discussion

In this study, we reported that melatonin administration could attenuate the right ventricular systolic pressure and mitigate the thickness of pulmonary arteriolar wall in the chronically hypoxic rats. In addition, the melatonin treatment could alleviate the oxidative stress and reduce the mRNA expression of pro-inflammatory mediators in the lung tissues in chronic hypoxia. Moreover, melatonin could increase the phosphorylated level at ser1177 position for the activation of eNOS activity. These findings are clinically relevant because pulmonary hypertension is observed in many pulmonary disorders including COPD, interstitial lung disease, sleep-disordered breathing, acute mountain sickness at high altitude, and also in children with congenital heart disease, in which the common cause is chronic exposure to hypoxia. Our results are also implicative on the prophylactic usage of antioxidant melatonin in these pulmonary disorders because oxidative stress and inflammation are believed to be involved in the pathogenesis of CH-induced vascular remodeling leading to pulmonary hypertension [5].

Consistent with findings in previous studies [24,26], we found that chronic hypoxia induced a significant elevated level of lipid peroxidation in the lungs, which was mitigated by the melatonin treatment. It has been shown that hypoxia increased the levels of lipid peroxidation in pulmonary endothelial cells and also the plasma glutathione disulfide in the animal [27,28]. In addition, hypoxic exposure or ROS could also induce oxidative burst in macrophages [29]. Thus, chronic hypoxia could increase the ROS generation resulting in oxidative stress. This could lead to an increase in the synthesis of platelet activating factor and pro-inflammatory cytokines that contribute to the pulmonary hypertension [29,30]. Indeed, hypoxia could activate pro-inflammatory pathways by increasing the expression of inflammatory cytokines and mediators [31,32], which in turn increase the expression of adhesion molecules [33,34]. As a result, progenitor cells could be recruited and migrated into pulmonary vascular walls for the medial thickening [5]. Melatonin is well known for its potent free radical scavenging ability and its high lipophilicity [35], which readily reduces the level of oxidative stress and this could explain the alleviated pulmonary hypertension in the melatonin-treated hypoxic animal.

Chronic hypoxia and its consequential increases in free radicals [36] induce the translocation of NF-κB to the nucleus and increase the NF-κB transcriptional activity. Activated NF-κB stimulates the synthesis of pro-inflammatory cytokines TNFα and interleukins and the subsequent expression of inflammatory mediators COX-2 and iNOS and also adhesion molecules including vascular cellular adhesion molecule-1, intercellular adhesion molecule-1 and E-selectin [36,37]. In addition, hypoxia changes the surface coagulant properties of endothelial cells by inducing procoagulant activity such as tissue factors [38] which are regulated by NF-κB [39]. In this regard, melatonin possesses multiple properties against inflammation, which regulates the expression of pro-inflammatory cytokines and inflammatory mediators probably through the negative modulation of the NF-κB activity [40]. In fact, we found that melatonin normalized the upregulated TNFα, COX-2 and iNOS levels in the hypoxic group. In addition, melatonin was found to decrease the expression of NF-κB in cultured cells and animal disease models [41,42,43]. Thus, it may reduce the CH-induced mRNA expression of pro-inflammatory cytokines and inflammatory mediators through the regulation of NF-κB expression.

We found an increased eNOS expression in the lungs in chronic hypoxia, which is consistent with previous findings [11,12,13,14,15,16]. The upregulation of the eNOS expression was considered a compensatory response to hypoxia, which is probably mediated by ROS [15,16]. However, the NO production is still limited by the lowered oxygen tension since the molecular oxygen is an essential substrate for the NO production by NOS. The half-maximal effective concentration for NO production in endothelial cells was found to occur at 38 Torr of oxygen, which is the normoxic level in pulmonary vasculature [44]. In hypoxia, an estimate of 35% reduction in the oxygen level could significantly lower the eNOS activity. In addition, the lowered NO levels in hypoxia may also be due to the formation of peroxynitrite because the NO level was increased by antioxidants, such as Cu/Zn superoxide dismutase and reduced glutathione [45]. Consequently, the lowered NO availability significantly contributes to the increased vasoconstriction and also the proliferation of smooth muscle cells in chronic hypoxia [46]. In this context, we found that the melatonin treatment significantly increased the level of phosphorylated eNOS (ser1177) in chronic hypoxia, which could elevate the NO production. This mechanism could explain the effects of melatonin on the RVSP probably with a lowered pulmonary vascular resistance and also on the vascular remodeling probably with a decreased proliferation of smooth muscle cells. The eNOS phosphorylation is mediated by several kinases including protein kinase A, protein kinase G, AMP-activated protein kinase and protein kinase B (Akt). Reports have shown that melatonin activated Akt activities [47,48,49]. Thus it is likely that melatonin may increase eNOS phosphorylation by activating the Akt pathway. Alternatively, potential effects of melatonin on the eNOS activation could be mediated by an increase in the endothelial intracellular Ca^2+^ level [50]; also a stabilization of the homodimerized form of eNOS with a lowered ROS level [51]. Consequently, the increase in eNOS activity could elevate the NO production and lower the pulmonary vasoconstriction, although the cardiac output and pulmonary vascular resistance were not determined in this study. In addition, the receptor-mediated effect of melatonin in pulmonary vessels may alter the vasoreactivity to ligands, although it could be relatively less because the antihypertensive effect of melatonin is known to be inhibited by chronic hypoxia [52].

## 4. Material and Methods

### 4.1. Hypoxic Treatment on Rats

Animal care and experimental protocol for this study (CULATR 2595-11, 09/01/2012) were approved by the Committee on the Use of Live Animals in Teaching and Research of The University of Hong Kong. The Laboratory Animal Unit of The University of Hong Kong is fully accredited by the Association for Assessment and Accreditation for Laboratory Animal Care (AAALAC International). Healthy Sprague-Dawley rats age 28 days (ca. 150 g weight) were divided into three groups, namely normoxic control (Nx) and hypoxic groups given either vehicle (CH) or melatonin (*n* = 6). While the Nx rats were maintained in room air, hypoxic rats were kept in acrylic chambers for normobaric hypoxia in the same room and had free access to water and regular chow. The oxygen fraction inside the chamber was maintained at 10% flushed with nitrogen and room air. The desired oxygen content was established by a mixture of room air and nitrogen which was regulated and monitored by an oxygen analyzer (Vacumetrics Inc., Ventura, CA, USA). The chamber was opened daily for 30 min for the drug administration and regular maintenance. The rats were exposed to hypoxia for 14 days and then were immediately used in experiments after taking out of the chambers.

### 4.2. Drug Preparation

Melatonin (Sigma, St. Louis, MO, USA) solution was prepared freshly before injection by dissolving the indoleamine in absolute ethanol and further dilution with normal saline; the final concentration of ethanol was 2%. Melatonin in 10 mg/kg body weight or vehicle (2% ethanol in normal saline) was administered intraperitoneally each day 30 min before hypoxic exposure.

### 4.3. Measurements of the Right Ventricular Systolic Pressure and Heart Weights

After the rat was anesthetized with pentobarbital sodium, a single-lumen catheter was inserted into the right ventricle at which the position of the catheter was judged by the waveform of the pressure signal. The right ventricular systolic pressure was recorded with a chart recorder (Lectromed, St. Ouen, Jersey, UK). The rats were decapitated and the hearts were quickly removed. The weights of right ventricles and left ventricles and the septa were determined.

### 4.4. Histological Analysis

The left lung was embedded in paraffin, sectioned (5 μm thick), and processed with Verhoeff/van Gieson staining to evaluate the muscularization of small resistance vessels (diameters of smaller than 50 μm and of between 50 and 100 μm) These sections were captured into digital images with a CCD JVC camera using a Zeiss Axiophot microscope at 40× magnification. The images were then analyzed by Leica QWIN Image Analyzer (Microsystems Ltd., Milton Keynes, Cambridge, UK) for measuring the thickness of the vascular layer of smooth muscle cells and also the diameter of the vessels.

### 4.5. Measurement of Malondialdehyde (MDA) Level

The MDA level was determined using a BIOXYTECH LPO-586™^®^ kit (OxisResearch, Portland, OR, USA). The reaction product was measured spectrophotometrically at 586 nm. Standard curves were constructed with 1,1,3,3-tetraethoxypropane as a standard. The MDA concentration (μM) in the lung was normalized to wet tissue weight (mg) and expressed as μmol/mg.

### 4.6. Western Blotting

Total protein was extracted by homogenizing tissue sample in RIPA buffer with protease inhibitor cocktail (Sigma) followed by centrifugation at 13,000× *g* for 15 min. Protein samples (30 mg per lane) were separated by 10% SDS-PAGE and transferred to PDVF membrane. Polyclonal antibodies eNOS, p-eNOS (Ser 1177) and monoclonal antibody β-actin were purchased from Santa Cruz Biotechnology Inc. (Santa Cruz, CA, USA). The PVDF membrane was blocked with 5% milk in Tris-buffered saline-Tween (0.05%) (TBST-milk) and incubated with primary antibody (total eNOS: 1/1000; p-eNOS (Ser 1177): 1/1000; β-actin: 1/10,000) for 1 h. The nitrocellulose membranes were washed with TBST solution and incubated with horseradish peroxidase-conjugated second antibody (1/10,000) for 1 h. The reaction was visualized by chemiluminescence with ECL Plus Western Blotting Detection System (Amersham, Buckinghamsire, UK). Films were exposed and analyzed by using Image J software (National Institutes of Health, Bethesda, MD, USA). The molecular weight of the band for eNOS or p-eNOS was determined at 140 kD and was at 43 kD for β-actin. Results were expressed in relative optical density.

### 4.7. Semi-Quantitative Reverse Transcription Polymerase Chain Reaction (RT-PCR)

Total RNA from the homogenate of the lung sample was extracted with Trizol reagents (Invitrogen Life Technologies, Carlsbad, CA, USA). Two micrograms of RNA was reverse transcribed into cDNA with Superscript™ first strand synthesis system (Invitrogen Life Technologies) for RT-PCR. PCR was done in RoboCycler (Stratagene, La Jolla, CA, USA) with Taq polymerase, AmpliTaq Gold™ (Roche Moelcular Systems, Pleasanton, CA, USA). Target genes for amplification and their primer sets and thermal conditions are listed in Table 1. The thermal cycles and annealing temperature for each target gene were tested with different cycle number and amount of RNA to confirm the optimization of the amplified product in the linear phase.

### 4.8. Statistical Analysis

Results are presented as means ± S.E.M. and statistical analyses between groups are one-way analysis of variance (ANOVA) with post-hoc Dunnett’s multiple comparison test for multiple comparison. Statistical significance was considered at *p* < 0.05.

## 5. Conclusions

In conclusion, melatonin attenuates pulmonary hypertension and antagonizes the oxidative injury. Thus, melatonin mitigates the inflammatory cascade induced by oxidative stress and the vascular remodeling. In addition, the regulation of eNOS phosphorylation by melatonin could play important roles in restoration of NO production and in controlling the vasodilatatory and proliferative responses of pulmonary arterioles in chronic hypoxia.

## Figures and Tables

**Table 1:** Primer sequences and reaction conditions for semi-quantitative reverse transcription polymerase chain reaction (RT-PCR).

Name	Sequence	Product Size (bp)	Annealing Temperature (T °C)	No. of Cycles (X)
COX-2	F: 5′-gtgttgacgtccagatcaca-3′ R: 5′-agtatgagcctgctggtttg-3′	662	58	28
iNOS	F: 5′-tagaggaacatctggccagg3′ R: 5′-tggccgacctgatgttgcca-3′	255	58	33
TNFα	F: 5′-atgagcacagaaagcatgatc-3′ R: 5′-tacaggcttgtcactcgaatt-3′	276	50	35
β-actin	F: 5′-agccatgtacgtagccatcc-3′ R: 5′-ctctcagctgtggtggtgaa-3′	228	56	28

The primer sets, annealing temperature (T °C) and the number of thermal cycles (X), for each target gene are listed above. Specific genes were amplified following the thermal conditions of 95 °C (15 min), followed by X cycles of 95 °C (1 min), T °C (1 min), 72 °C (1 min), and then 72 °C (10 min).
